# Pathology students’ perceptions of virtual learning: A case study of students in Saudi Arabia

**DOI:** 10.1371/journal.pone.0307150

**Published:** 2024-08-12

**Authors:** Nawal Hamdan Almohammadi

**Affiliations:** Department of Basic Medical Sciences, Faculty of Medicine, Taibah University, Madinah, Saudi Arabia; University of Alcala Faculty of Medicine and Health Sciences: Universidad de Alcala Facultad de Medicina y Ciencias de la Salud, SPAIN

## Abstract

**Background:**

Pathology laboratory classes are traditionally conducted using a conventional light microscope. The Coronavirus Disease 2019 (COVID-19) pandemic and recent technological advances necessitated remote learning through online classes using virtual slides (VS) instead of glass slides (GS).

**Aim:**

The purpose of this study was to gauge the perception of learning pathology using virtual slides (VS) as opposed to glass slides (GS) for medical students in Saudi Arabia. This study would help modify teaching methods with the advancement of the application of newer methods in online teaching.

**Methods:**

This two-phased study evaluated learning outcomes and perceptions in pathology online education for medical students. Using a questionnaire, Phase one analyzed second and third-year students’ perceptions of the teaching methods after an online pathology course. Phase Two assessed the learning outcomes of third-year students during online practical sessions using a pretest and post-test design. Statistical data were collected using a simple additive approach. Statistical tools were used to determine the factors affecting students’ perceptions.

**Results:**

The accessibility of VS at any possible time, location, or device was the most advantageous trait of virtual learning (mean = 2.94±0.9). Students agreed the least with virtual slides as the only optimal method of learning pathology (mean = 2.25±0.9). Most enjoyed the virtual lab experience (51.7%) but still prefer both laboratory-GS and virtual-VS classes (83.5%).

**Conclusions:**

VS had the benefit of accessibility and efficiency. The acceptance of VS was significantly affected by the orientation prior to the online class. Findings showed that VS cannot completely replace GS and more aspects such as technical difficulties and prior VS experience should be explored.

## Introduction

Wuhan City, China reported the first case of coronavirus disease 2019 (COVID-19) on December 13, 2019. Since COVID-19 initially emerged in China, the virus has evolved over four months and swiftly spread to other countries around the world, posing a global risk. Saudi Arabia reported its first COVID-19 case on March 2, 2020, and by March 11, nearly three months since the transmission of the infection, the World Health Organization (WHO) categorized COVID-19 as a worldwide pandemic [1, 2]. In compliance with government-mandated protocols and lieu of social distancing limitations, the Saudi Arabian government was quick to respond to the health crisis, imposing several preventative measures such as border measures, suspension of gathering activities, lockdowns, and curfews; educational institutions decided to continue the teaching and learning process in a safe and secure manner, so they announced virtual teaching and online assessment strategies for all universities across the country, including health sciences universities, in accordance with the WHO’s social distancing protocol to prevent the spread of COVID-19. In the United States of America (USA) and several other countries, classes were live-streamed, webinars were offered, and social media platforms such as Twitter, Zoom, and Google were used for tutorials [3, 4].

With the emergence of the COVID-19 pandemic, the disruption in medical education worldwide has had a significant impact on the delivery of lessons in all educational institutions, as the shift to a physically distanced learning environment has been observed; this included affecting the field of pathology classes; moreover, comparing pathology laboratory classes were taught in separate camps before COVID-19 [3]. Even before the pandemic, the field of pathology has advanced multiple digital pathology systems in recent years. Since its first use in the late 1960s, many versions of digital pathology have been utilized for clinical and educational reasons, with different digital pathology systems reported in the literature. Digital pathology systems are now being used to send virtual images to distant or remote locations for consultation or frozen section diagnosis, and a selective review of the literature has revealed a consistent trend of concordance between various forms of digital pathology and traditional light microscopy [5, 6]. Moreover, digital pathology using whole slide imaging (WSI) has also emerged as the digital pathology platform of choice for teaching in recent years. It has steadily gained a foothold in medical education, diagnosis, and training; both utilized in undergraduate and graduate medical education, primarily by leveraging stored study sets, teaching libraries and individual cases to monitor acquisition of new skills (eg, stain interpretation), to enhance didactic teaching, and to assess competency through slide examination/testing [6, 7]. It is also known as virtual slides (VS), which are digitally scanned glass slides provided in high-resolution image quality. The VS can be retrieved and reviewed by running specific software in a web browser, simulating the visual experience of viewing glass slides with a light microscope. This technology allows students to access digital slides remotely using smart devices or computer monitors instead of microscopes. Alternatively, a transition to a dynamic virtual microscopy (DVM) provides a conveniently available, physically distant, and cost-effective option for pathology education. Dynamic virtual microscopy platform is defined as one light microscope with a mounted digital camera for the educator, a digital camera, video conferencing software to stream a slide image to the learner(s), and one computer per participant. A key advantage of virtual slide viewers is their ability to replicate the functionality of traditional microscopes by offering a range of magnification options. This allows users to zoom in on specific regions of interest for closer examination while retaining the ability to view the slide in its entirety at lower magnifications. Also, with the recent advances in technology and digitalization of learning, most clinical schools have embraced virtual microscopy (VM) into their educational programs [5–7].

Numerous studies provided excellent outcomes with virtual slides for undergraduate students and postgraduate training in pathology, diagnostic surgical histopathology, and cytopathology. A 2023 cross sectional study aimed to determine the rewards and struggles of online pathology learning during the pandemic at the Al-Qunfudah Medical College, Kingdom of Saudi Arabia wherein results showed that the students accepted the online platform as a tool for studying pathology online. Moreover, it is emphasized that extensive training provided to teachers can significantly increase the support given to students during online teaching [8]. In contrast, reports of disadvantages for students and professors in many health sciences courses, particularly medical sciences students have also been noted due to the impact on laboratory and clinical teaching modes in medical institutions and hospitals during COVID-19 [9]. One 2023 study aimed to evaluate distance learning teaching method of the Pathology College with reference to the learner’s point of view in which results showed drawbacks of the approach included a lack of face-to-face connection with lecturers and colleagues, as well as the occurrence of technological issues that periodically affected the smooth operation of the sessions. In the survey, 73% of participants believed that face-to-face courses were presented more clearly than online courses.

Given the unique nature of the field of pathology, the importance of analyzing slides in a traditional histology setting in acquiring diagnostic skills, and the sudden replacement of face-to-face training devices with digital means was an unprecedented experience, as medical students had to quickly adapt to learning exclusively online. Due to the noted restrictions in the educational field, educational innovation has been required to keep teaching pathology in optimal means. A trend toward teaching pathology through dynamic virtual microscopy provides an easily available and cost-effective option. Thus, its impact on the pathology curriculum was raised.

Hence, in this study, we explored how medical students perceive and perform when learning pathology through online classes using virtual slides. It aimed to understand students’ perspective on the transition, potential improvements, and their performance compared to traditional glass slides. In addition, it can help educators refine online pathology classes and inform future teaching methods.

## Materials and methods

### Ethics statement

This research project was approved by the Research Ethics Committee (CM-REC) at Taibah University College of Medicine (Study ID: 017–1442). This ensured adherence to the ethical principles outlined in the Declaration of Helsinki. All participants were adults (above the age of 18) and provided written informed consent after being fully informed about the study’s purpose, procedures, potential risks and benefits, and their right to withdraw at any point. A detailed participant information sheet was provided, and participants were given ample opportunity to ask questions before consenting and participating. Participants’ privacy was safeguarded by anonymizing all collected data.

### Study design and participants

This two-phased study evaluated learning outcomes and perceptions in pathology online education for medical students conducted from 03/02/2021 to 03/04/2021. Using a self-administered questionnaire, Phase One analyzed the perceptions of second and third-year students of the teaching methods after an online pathology course. In Phase Two, learning outcomes of third-year students during online practical sessions were assessed using a pretest and post-test multiple-choice questions design. A total of two hundred seventy-one medical students (excluding 4 who did not consent) participated in the study.

### Teaching methods

Virtual slide sessions were conducted online using PowerPoint presentations. Supplementary materials included a PDF handout, a workbook, and pre/post-tests. Slide access was secure and open for external participants. Students were able to view slides through any web browser using Flash technology and server software (WebScope and ImageScope). This virtual slide viewers allowed students to zoom in on specific regions of interest and to view the slide in its entirety at lower magnifications. Each presentation had a unique link provided to students.

### Perception assessment questionnaire

Student demographics (academic year, gender, age) were collected. After the online pathology classes, a survey assessed student impressions of the virtual class. Survey statements were positively worded, highlighting the potential of virtual slides for accessibility, viewing experience, and learning compared to glass slides. Students rated their perceptions of various aspects of virtual slide learning via a 5-point Likert scale (5 = strongest agreement, 1 = strongest disagreement). The survey also included questions about the presentation method, supplementary materials, and how virtual slides impacted learning and knowledge acquisition.

### Statistical analysis

This research was analyzed and visually presented using International Business Machines Corporation Statistical (IBM SPPS) ver. 23 (IBM Corp., Armonk, N.Y., USA) and using GraphPad Prism version 8 (GraphPad Software, Inc., San Diego, CA, USA), respectively. The features of the research variable were defined using a simple descriptive statistic in the form of counts and percentages for the categorical and nominal variables, while consistent factors were introduced by mean and standard deviations. Items related to the perception of VS against GS were answered using the Likert-style scale and were scored using a basic addition strategy and was changed over to a 100-point scale. Also, a Reliability Analysis was used with a model of Alpha (Cronbach, > 0.7) to investigate the measurement scales’ features, the items that make up the scales, and the normal inter-item correlation. To compare the student’s perception of VS against GS with the demographical data and other predictors, an independent *t-*test was used for two-group means and for more than two groups- a One-way Analysis of Variance (ANOVA) with Least Significant Difference (LSD) as a post hoc test was used. To correlate variables which, both represented by means, a Pearson’s correlation coefficient was used. The normal distribution assumption was used in these tests. Finally, the null hypothesis was rejected if the p-value was less than 0.05.

## Results

In order to assess students’ perceptions of teaching methods (Phase One), the questionnaire analysis reported data from a total of two hundred seventy-one medical students (including 4 who did not consent) leading to 267 medical students, 167 (62.5%) of whom are second-year students, and the remaining are third-year students (37.5%). The majority (n = 136, 50.9%) of the students were male, and the mean age of the population (n = 267) was 20.21 years of age. The overall response rate was 100%.

**Table 1** presents the percentage of student agreement with specific statements from the questionnaire. All statements were positively worded in favor of virtual slides, and they also evaluated the participant’s perception of virtual slides. The majority of the students responded that they ‘agree’ in 11 out of 13 questions (84.6%), particularly that virtual slides have a better viewing, accessibility, and learning experience than glass slides. However, most of the students were undecided, neither agreeing nor disagreeing, with having the virtual slides as the only optimal method of teaching pathology 111 (41.6%) and with having the virtual class increase teacher and student interaction 94 (35.2%).

**Table 1:** Student evaluation of virtual slide against glass slides post virtual online class (N = 267, 100%).

Question Item	Totally Disagree	Disagree	Neither agree nor disagree	Agree	Totally Agree
1. I prefer Virtual slide to the Glass slide	16 (6%)	36 (13.5%)	78 (29.2%)	**92 (34.5%)**	45 (16.9%)
2. The quality of the image of virtual slides is better than Glass Slide	10 (3.7%)	28 (10.5%)	90 (33.7%)	**92 (34.5%)**	47 (17.6%)
3. The identification of cells and structures in Virtual Slide is better than Glass Slide	6 (2.2%)	24 (9%)	82 (30.7%)	**113 (42.3%)**	42 (15.7%)
4. Navigation with Virtual Slide viewer is easier than with Glass Slide	4(1.5%)	17 (6.4%)	89 (33.3%)	**119 (44.6%)**	38 (14.2%)
5. Virtual slide is easy to use and effective for the practical session.	3 (1.1%)	22 (8.2%)	77 (28.8%)	**128 (47.9%)**	37 (13.9%)
6. I liked the possibility to access the images anywhere and at any time	3 (1.1%)	11 (4.1%)	54 (20.2%)	**130 (48.7%)**	69 (25.8%)
7. I can quickly access virtual slides from any devise	2 (0.7%)	23 (8.6%)	60 (22.5%)	**120 (44.9%)**	62 (23.2%)
8. Virtual slide permits learning in less time	3 (1.1%)	9 (3.4%)	88 (33%)	**117 (43.8%)**	50 (18.7%)
9. I succeeded in covering session ILOs through using Virtual Slide	5 (1.9%)	34 (12.7%)	88 (33%)	**109 (40.8%)**	31 (11.6%)
10. Virtual Slide improve my learning	3 (1.1%)	18 (6.7%)	86 (32.2%)	**122 (45.7%)**	38 (14.2%)
11. I recommend using Virtual Slide session in my upcoming practical pathology lesson	4 (1.5%)	24 (9%)	75 (28.1%)	**119 (44.6%)**	45 (16.9%)
12. Virtual slide only is optimal for pathology learning	5 (1.9%)	49 (18.4%)	**111 (41.6%)**	78 (29.2%)	24 (9%)
13. Virtual slide can improve student’s interactions and improve the interaction with the teachers when compared with glass slide	16 (6%)	27 (10.1%)	**94 (35.2%)**	91 (34.1%)	39 (14.6%)

**Fig 1:**
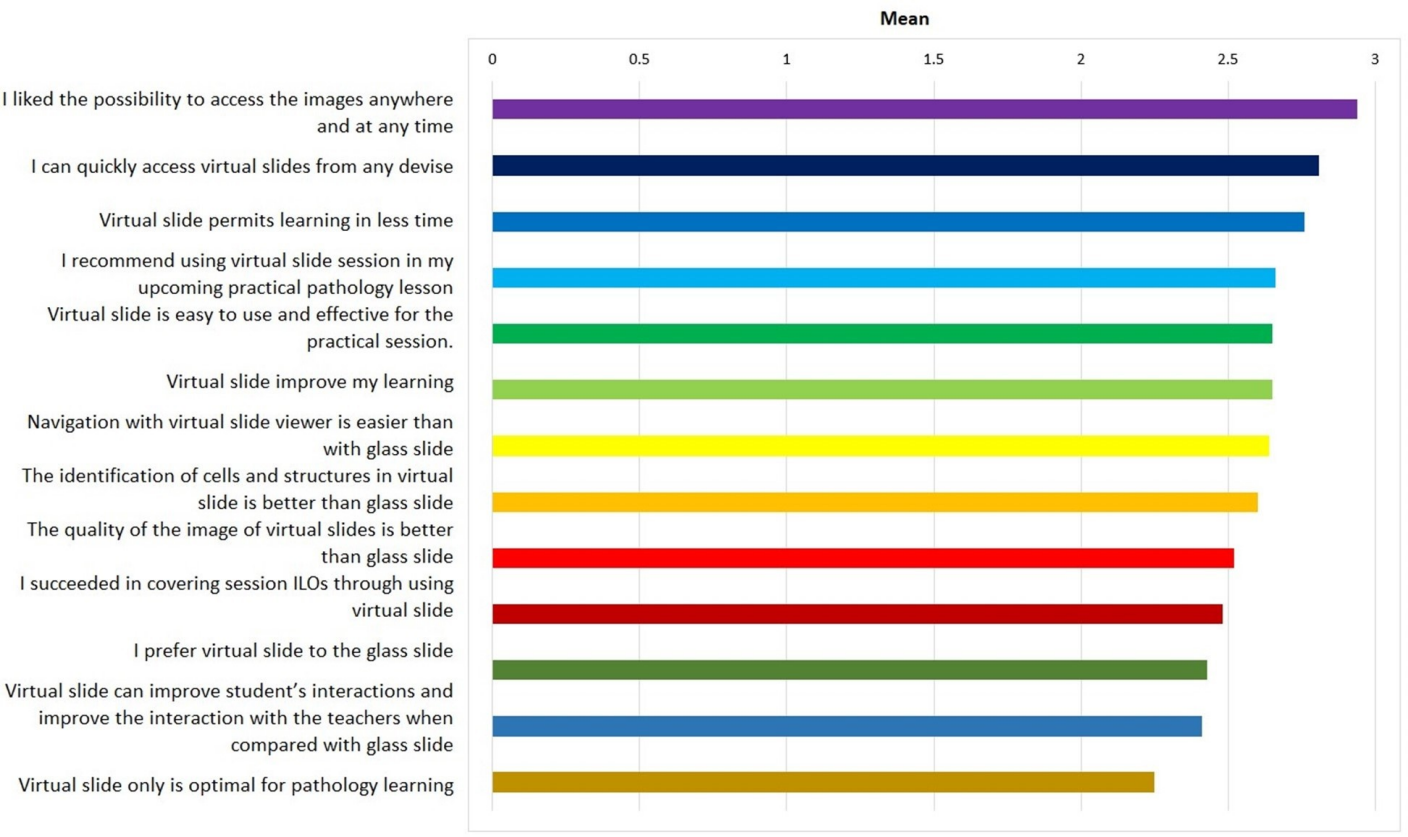
Student’s perception on virtual slides in point scale.

For better data representation, results from the Likert scale’s questions in **Table 1** were converted to a point scale in **Table 2**, with zero indicating total disagreement and four indicating total agreement. Furthermore, data in **Table 2** is represented in **Fig 1**. All the individual question items had a mean above 2, indicating a positive perception of virtual slides compared to the glass slide. Across all the year levels, the accessibility of the images **‘**anywhere and at any time**’** (2.94±0.9) was found to be the most significant feature that favors virtual slides over glass slides. The ability to access the virtual slide using any device (2.81±0.9) and the convenience of learning in a shorter amount of time (2.76±0.8) were also received in high positivity by the students. On the other hand, the perception that virtual slides were the only optimal method of learning (2.25±0.9) and the increased interaction among students and teachers while using virtual slides received the lowest agreement (2.41±1.0).

**Table 2:** Student’s perception on virtual slides from Likert’s scale presented in point scale.

Question Item	Mean±SD (Min = 0, Max = 4)
1. I prefer virtual slide to the glass slide	2.43±1.1
2. The quality of the image of virtual slides is better than glass slide	2.52±1.0
3. The identification of cells and structures in virtual slide is better than glass slide	2.60±0.9
4. Navigation with virtual slide viewer is easier than with glass slide	2.64±0.9
5. Virtual slide is easy to use and effective for the practical session.	2.65±0.9
6. I liked the possibility to access the images anywhere and at any time	**2.94±0.9**
7. I can quickly access virtual slides from any devise	2.81±0.9
8. Virtual slide permits learning in less time	2.76±0.8
9. I succeeded in covering session ILOs through using virtual slide	2.48±0.9
10. Virtual slide improve my learning	2.65±0.8
11. I recommend using virtual slide session in my upcoming practical pathology lesson	2.66±0.9
12. Virtual slide only is optimal for pathology learning	2.25±0.9
13. Virtual slide can improve student’s interactions and improve the interaction with the teachers when compared with glass slide	2.41±1.0

Reliability statistics were done to determine the agreeability of the responses of all participants with the acceptance of >0.7 as Cronbach’s Alpha. For the 13 question items regarding the perception on virtual slides, Cronbach’s Alpha was 0.875, and the agreeability of the answers of all participants was 87.5%. Answers obtained from the 5-point Likert Scale were also converted to a hundred-point scale. Data showed that the answers of 267 participants had a mean value of 65.00±14.7, where the minimum value was 32.69 and the maximum was 100. The mean value was 65.00±14.7.

**Table 3** shows the evaluation of predictors of students’ perception on virtual slides wherein there was a significantly higher acceptance **(p = 0.011)** among students who felt they had adequate orientation (68.12 ± 14.9) as compared to students who did not have enough orientation (62.82 ± 14.7) or were unsure if they had enough orientation (62.58 ± 13.8) about online teaching before the virtual class. On the other hand, evaluation of predictors of students’ perception on virtual slides showed that academic year (p = 0.070), gender (p = 0.259), and presence of anxiety before the online class (p = 0.457) had no significant relation with the acceptance.

**Table 3:** Factors affecting student’s acceptance and perception on virtual slides.

Variables		Total	Virtual slide	p-value
Academic year	2	167	63.74 ± 14.0	0.070
	3	100	67.10 ± 15.6	
Gender	Male	136	64.00 ± 15.5	0.259
	Female	131	66.03 ± 13.7	
Did you have sufficient orientation about online teaching before attending the online Pathology course?	Yes	114	68.12 ± 14.9^A^	**0.011** ^**a**^
	No	57	62.82 ± 14.7^B^	
	Maybe	96	62.58 ± 13.8^B^	
Did you have any anxiety regarding the Pathology course before attending online teaching?	Yes	93	65.96 ± 16.1	0.457
	No	106	65.35 ± 13.2	
	Maybe	68	63.12 ± 14.9	

^a^- significantly correlated using one way ANOVA, ^A^-significantly different from ^B^

An assessment of the overall virtual online class is presented in **Table 4**. The majority of the 267 participants felt that the virtual pathology lab somewhat reinforced the lecture class (n = 178, 66.7%) and enjoyed the virtual class while learning pathology-related material (n = 138, 51.7%). More than half (n = 141, 52.8%) of the participants learned moderately sufficient new information from the virtual pathology class. Moreover, 43.1% (n = 115) of the students also found PowerPoint and PDF handouts for virtual sessions helpful. Still, students prefer learning through combined virtual and glass slides (n = 223, 83.5%) and from both online and physical laboratories equally (n = 99, 37.1%).

**Table 4:** Overall assessment of the online virtual class- student’s learning and preferred teaching method.

Variables		Count	%
Total		267	100.0
To what extent do you feel that the Virtual pathology lab reinforced the information that you received in the lecture?	I do not feel that any of the information from the Virtual pathology lab reinforced the information we received in the lecture.	50	18.7
	I feel that the Virtual pathology lab somewhat reinforced the information we received in the lecture.	178	66.7
	I feel that the pathology virtual lab experience completely reinforced the information we received in the lecture.	39	14.6
To what extent do you feel that you learned new information from your experience with the Virtual pathology lab this academic year?	1 Insufficient	11	4.1
	2 Fairly sufficient	27	10.1
	3 Moderately sufficient	141	52.8
	4 Sufficient	66	24.7
	5 Abundant	22	8.2
What was your level of enjoyment as you went through the virtual pathology lab experience this academic year?	I enjoyed the Virtual pathology lab learning experience and felt it helped me learn the pathology related material.	138	51.7
	I did not enjoy the Virtual pathology lab learning experience but did feel that it helped me learn the pathology related material.	107	40.1
	I did not enjoy the Virtual pathology lab learning experience and did not feel that it helped me learn the pathology related material.	22	8.2
PPT presentation and PDF handout accompanied virtual session are useful	Totally Disagree	3	1.1
	Disagree	15	5.6
	Neither agree nor disagree	85	31.8
	Agree	115	43.1
	Totally Agree	49	18.4
Based on your experience, please describe how you believe your ideal pathology lab experience would be structured.	Glass slides only	18	6.7
	Combined glass slide and virtual slide	223	83.5
	Virtual slide only	26	9.7
Based on your experience, please describe how you believe your ideal pathology lab experience would be structured.	100% in lab	27	10.1
	75% in lab; 25% virtual	74	27.7
	50% in lab; 50% virtual	99	37.1
	25% in lab; 75% virtual	57	21.3
	100% virtual	10	3.7

In the phase two study, we assessed the extent of students’ learning through the online pathology class using a pre-and post-test done for three topics: obstructive renal diseases of kidneys and ureters, and benign prostatic hyperplasia, hepatitis, and liver cirrhosis, and neoplasia, and colorectal carcinoma. As seen in **Table 5**, it was observed that for the first topic of obstructive renal diseases of kidneys and ureters and benign prostatic hyperplasia, there was reduction in participation from 33 (64.7%) responses to only 18 responses (35.3%). A similar occurrence was noted for the basics of neoplasia and colorectal carcinoma practical session (66.9% to 33.1%). Only hepatitis and liver cirrhosis practical sessions received increased participation from students.

**Table 5:** Response rate of the students pre and post online pathology class examination.

Session	Period	Count	%
1. Obstructive Renal Diseases of Kidneys and, Ureters, and Benign Prostatic Hyperplasia	Pre	33	64.7
	Post	18	35.3
2. Hepatitis and Liver Cirrhosis Practical Session	Pre	59	46.8
	Post	67	53.2
3. Basic of Neoplasia and Colorectal Carcinoma Practical Session	Pre	109	66.9
	Post	54	33.1

The average of the correct answers from pre-and post-test of each topic was obtained and shown in **Table 6**. Mean scores of the pre and post-examination for the first topic decreased; however, the difference was not significantly varied. No significant increase was found in the scores of the third topic. Only in the topic of hepatitis and liver cirrhosis practical session, was there an observed slight but significant increase (p<0.001) in mean score from 3.36±1.0 pre-virtual class to 4.07±1.1.

**Table 6:** Average score of the students pre and post online pathology class examination.

Session	Total	Period		P value
		Pre	Post	
1. Obstructive Renal Diseases of Kidneys and, Ureters, and Benign Prostatic Hyperplasia	51	2.82 ± 1.4	2.50 ± 1.2	0.420
2. Hepatitis and Liver Cirrhosis Practical Session	126	3.36 ± 1.0	4.07 ± 1.1	**<0.001** ^**a**^
3. Hepatitis and Liver Cirrhosis Practical Session	163	1.96 ± 1.2	2.22 ± 0.8	0.109

^a^-significant using Independent *t*-test @<0.05 level.

In-depth analysis showed that more students got the correct answers in all five questions about hepatitis and liver cirrhosis after the class **Table 7**. Despite this, only questions 1 (79.7% to 92.5%) and 3 (61.0% to 88.1%) showed a significant increase from pre- to post-examination results (both p<0.001).

**Table 7:** Results of the pre and post examination of hepatitis and liver cirrhosis practical session.

Question Items	Total	Period		p-value
		Pre	Post	
Total	126	59(46.8%)	67(53.2%)	-
Points	126	3.36 ± 1.0	4.07 ± 1.1	<0.001^a^
1. This microscopic view from liver shows: (VS 1)	109	47(79.7%)	62(92.5%)	**0.035** ^**b**^
2. This microscopic view from liver shows: (VS 2)	85	37(62.7%)	48(71.6%)	0.286
3. This microscopic view from liver shows: (VS 3)	95	36(61.0%)	59(88.1%)	**<0.001** ^**b**^
4. What is the best description for the gross appearance shown here? (VS 4)	102	45(76.3%)	57(85.1%)	0.209
5. What is the best description for the gross appearance shown here? (VS 5)	80	33(55.9%)	47(70.1%)	0.098

^a^-significant using Independent *t*-test @<0.05 level.

^b^-significant using Chi-Square Test @<0.05 level.

In comparison, five of the question items for basic neoplasia and colorectal carcinoma had lower correct answers post-examination as compared to the exam prior to the online class **Table 8**. Moreover, in the remaining three questions, only two had a significantly better result. There was a significant increase in correct responses from 45.9% to 68.5% in question item 1 (p = 0.006) pre and post-test. Likewise, more students scored significantly (p = 0.002) in question item 8 (56.9% to 81.5%).

**Table 8:** Results of the pre and post examination of basic of neoplasia & colorectal carcinoma practical session.

Question Items	Total	Period		p-value
		Pre	Post	
Total	163	109(66.9%)	54(33.1%)	-
Points	163	1.96 ± 1.2	2.22 ± 0.8	0.109
1. What is the best diagnosis for the gross appearance of colon shown here? (VS 1)	87	50(45.9%)	37(68.5%)	**0.006** ^**a**^
2. What is the best diagnosis for the gross appearance of the colon shown here? (VS 2)	21	16(14.7%)	5(9.3%)	0.331
3. What is the best diagnosis for the gross appearance of the colon shown here? (VS 3)	14	11(10.1%)	3(5.6%)	0.331
4. What is the best diagnosis for the gross appearance of the colon shown here? (VS 4)	28	23(21.1%)	5(9.3%)	0.059
5. This microscopic view from colon shows: (VS 5)	18	14(12.8%)	4(7.4%)	0.297
6. This microscopic view shows: (VS 5)	50	30(27.5%)	20(37.0%)	0.215
7. This microscopic view shows: (VS 6)	10	8(7.3%)	2(3.7%)	0.363
8. This microscopic view shows: (VS 6)	106	62(56.9%)	44(81.5%)	**0.002** ^**a**^

^a^-significant using Chi-Square Test @<0.05 level.

## Discussion

This current study is a comprehensive analysis of the perceptions of medical students in Saudi Arabia using virtual learning in the pathology field. Several main points from the results of the study are assessed below.

Prior to this study, the participants were accustomed to being in a physical laboratory setup using a light microscope and viewing a single glass slide at a time. For this research, students were initially assessed for their knowledge about pathology topics using pre ‐ test. Afterward, an online pathology class was conducted using virtual slides supplemented with PowerPoint and PDF handouts. After the class, the students were again evaluated for the reinforced or new knowledge gained from the recent class (post-test). Additionally, the students were asked about their overall perception of the use of virtual slides (VS) instead of glass slides (GS) using questionnaire.

The current study showed reported data from 267 medical students, 167 (62.5%) of whom are second-year students, and the remaining are third-year students (37.5%). The majority (n = 136, 50.9%) of the students were male, and the mean age of the population was 20.21 years of age. Responses from the questionnaire revealed that majority of the students responded that they ‘agree’ in 11 out of 13 questions (84.6%), particularly that virtual slides have a better viewing, accessibility, and learning experience than glass slides. For better data representation, results from the Likert scale’s questions were also converted to a point scale in **Table 2**, with zero indicating total disagreement and four indicating total agreement. Furthermore, all the individual question items had a mean above 2, indicating a positive perception of virtual slides compared to the glass slide. Across all year levels, the accessibility of the images **‘**anywhere and at any time**’** (2.94±0.9) was found to be the most significant feature that favors virtual slides over glass slides. The ability to access the virtual slide using any device (2.81±0.9) and the convenience of learning in a shorter amount of time (2.76±0.8) were also received in high positivity by the students. This is in line with a 2023 descriptive cross-sectional study wherein pathology residents were asked for their point of view as to distance learning approach wherein results showed that concerning virtual pathology slides, participants found that acquiring diagnostic skills via the virtual slide websites was better than learning in the traditional setting in 53.8% of cases [10]. The main two reasons found in the study were: residents had more time to analyze virtual slides than in the traditional histology setting (42.9%) and residents had the ability to view virtual slides at any time (35.7%). The main strength observed by the participants was the flexibility of learning schedules and locations (73.1%).

On the other hand, most of the students were undecided, neither agreeing nor disagreeing in the current study, with having the virtual slides as the only optimal method of teaching pathology 111 (41.6%) with mean±SD (2.25±0.9) and with having the virtual class increase teacher and student interaction 94 (35.2%) with mean±SD (2.41±1.0). Also, from the 2023 descriptive cross-sectional study, drawbacks from using virtual approach in pathology were noted in which dependence on technical means (42.3%), the lack of interactivity with colleagues (26.9%) and the lack of interactivity with teachers (26.9%) were observed [10]. Also found in a 2023 study where they aimed to determine the rewards and struggles of online pathology learning during the coronavirus disease 2019 (COVID-19) pandemic, students reported that social distancing disturbed their interpersonal communications during the pandemic and hindered their peer-learning opportunities [8]. In accordance with this, in a 2021 survey, it was observed that virtual microscopy also came with significant disadvantages wherein results showed image focus and resolution, distortion of white balance, image lag time, and discordant fields-of view were among the main difficulties for both faculty and medical trainees [5]. Further, a 2022 study found that the lack of hands-on laboratory activities such as tissue grossing and operating the light microscope were among the drawbacks of using virtual pathology education [11].

Evaluation of predictors of students’ perception on virtual slides were also assessed in the current study in which results showed that there was a significantly higher acceptance (p = 0.011) among students who felt they had adequate orientation (68.12 ± 14.9) as compared to students who did not have enough orientation (62.82 ± 14.7) or were unsure if they had enough orientation (62.58 ± 13.8) about online teaching before the virtual class. Among the factors affecting students’ acceptance and perception of virtual slides, the question of whether participants had sufficient orientation about online teaching before attending the online Pathology course was found to be significant in terms of perception of virtual slide with a p-value of 0.011 using one-way ANOVA. In line with a 2021 cross-sectional descriptive study, e-learning readiness of the students of the following institute were assessed in which results showed that more than 73% (n = 84) of the participants have acknowledged the present form of online classes to be the best available option in COVID-19 lockdown and most of them are adapted to e-classes. Students showed that they are ready in terms of attitude to attend e-learning classes given prior orientation and were found to be technologically self-sufficient after 1 month of attending e-classes and students [12]. Further seen in the results, academic year (p = 0.070), gender (p = 0.259), and presence of anxiety before the online class (p = 0.457) had no significant relation with the predictors of students’ perception on virtual slides.

Overall assessment of virtual online class was also assessed in the current study wherein results revealed that the majority of the participants noted that the virtual pathology lab somewhat reinforced the lecture class (n = 178, 66.7%) and enjoyed the virtual class while learning pathology-related material (n = 138, 51.7%). More than half (n = 141, 52.8%) of the participants learned moderately sufficient new information from the virtual pathology class. Moreover, 43.1% (n = 115) of the students also found PowerPoint and PDF handouts for virtual sessions helpful. Still, students prefer learning through combined virtual and glass slides (n = 223, 83.5%) and from both online and physical laboratories equally (n = 99, 37.1%). This is in accordance with a recent study where they focused on evaluating e-learning module approach in pathology courses in India where results showed that e-learning is typically viewed as a supplement to traditional classroom-based instruction [13]. Moreover, two studies on pathology courses were also conducted in the USA, where the first study aimed to assess medical students’ perceptions of learning in an advanced clinical pathology course offered in a remote learning environment. The overall good feedback from students demonstrated that many areas of anatomic pathology have been successfully adapted to the e-learning environment. The second study, undertaken at the University of Iowa, compared pathology teaching before and during the pandemic based on the curriculum. Student reaction was largely favorable, and end-of-course surveys indicated a preference for live streaming instruction in small groups [14, 15].

In the phase two of the study, we assessed the extent of students’ learning through the online pathology class using a pre-and post-test done for three topics: obstructive renal diseases of kidneys and ureters, and benign prostatic hyperplasia, hepatitis, and liver cirrhosis, and neoplasia, and colorectal carcinoma. Results show that of the three-course topics, only VS about hepatitis and liver cirrhosis had a significant improvement (p<0.001) using independent t-test at <0.05 level on mean test scores. In depth analysis showed that, of the five questions about hepatitis and liver cirrhosis, questions 1 (79.7% to 92.5%) and 3 (61.0% to 88.1%) showed a significant increase from pre- to post-examination results (both p<0.001). Based on these, it can be inferred that despite the significant improvement in learning through virtual slides, the participants of this study did not, to any great extent, increase their correct answer scores. Moreover, in comparison, five of the question items for basic neoplasia and colorectal carcinoma had lower correct answers post-examination as compared to the exam prior to the online class. In the remaining three questions, only two had a significantly better result. There was a significant increase in correct responses from 45.9% to 68.5% in question item 1 (p = 0.006) pre and post-test. Likewise, more students scored significantly higher (p = 0.002) in question item 8 (56.9% to 81.5%).

This study may be limited by its relatively small sample size of 267 participants which could potentially constrain its representativeness. A larger sample from multiple universities and regions is suggested. Longitudinal studies are essential for understanding the long-term effects of online education. Thus, having a more diverse sample of health sciences students would improve the study’s external validity. Furthermore, faculty training workshops should be held to develop online teaching practices and incorporate virtual tools to improve the quality of online education. Finally, it should be recognized that adjusting to new technologies requires time. Individual users may adapt quickly or slowly to the new way, and each may require varied degrees of technology assistance to develop their operating skills with the new system.

## Conclusion

In conclusion, this study gives information on health sciences students’ perception towards virtual learning during the COVID-19 pandemic in Saudi Arabia. Despite the participants’ specific issues and preferences, the data show widespread satisfaction and acceptance with online teaching and learning. Notably, aside from accessibility and efficiency, VS also minimally reinforced the student’s knowledge and allowed students to gain new information as evidenced by the results found in the two phase study conducted in which pre-and post-tests were done for three topics including obstructive renal diseases of kidneys and ureters, and benign prostatic hyperplasia, hepatitis, and liver cirrhosis, and neoplasia, and colorectal carcinoma.

Overall, the findings contribute to the continuing discussion about online education and highlight the importance of constant improvement and adaptation of teaching methods to meet students’ evolving expectations in the digital age. Furthermore, ideas for faculty training workshops seek to improve the quality of online education and ensure effective and interesting learning experiences for students participating in health sciences programs.
